# The genome sequence of the European mistletoe, *Viscum album* L. (Santalales: Viscaceae)

**DOI:** 10.12688/wellcomeopenres.24920.1

**Published:** 2025-09-15

**Authors:** Lucia Campos-Dominguez, Maarten J. M. Christenhusz, David Bell, Michael F. Fay, Michelle Hart, Peter M. Hollingsworth, Ilia J. Leitch, Sahr Mian, Kanae Nishii, Royal Botanic Gardens Kew, Royal Botanic Garden Edinburgh, Darwin Tree of Life Barcoding Collective, Wellcome Sanger Institute Tree of Life Management, Samples and Laboratory team, Wellcome Sanger Institute Scientific Operations: Sequencing Operations, Wellcome Sanger Institute Tree of Life Core Informatics team, Tree of Life Core Informatics collective, Darwin Tree of Life Consortium, Alex D. Twyford

**Affiliations:** 1 Center for Research in Agricultural Genomics, CRAG (CSIC-IRTA-UAB-UB), Campus UAB, Cerdanyola del Vallès, 08193, Barcelona, Spain; 2 The University of Edinburgh, Edinburgh, Scotland, UK; 3 Curtin University, Perth, Western Australia, Australia; 4 Royal Botanic Gardens Kew, Richmond, England, UK; 5 Royal Botanic Garden Edinburgh, Edinburgh, Scotland, UK; 6 The University of Western Australia, Perth, Western Australia, Australia

**Keywords:** Viscum album; European mistletoe; genome sequence; chromosomal; Santalales

## Abstract

We present a genome assembly from a female specimen of *Viscum album* (European mistletoe; Streptophyta; Magnoliopsida; Santalales; Viscaceae). The genome sequence has a total length of 94 261.04 megabases. Most of the assembly (98.67%) is scaffolded into 49 chromosomal pseudomolecules, including the B _1_ chromosome. This assembly was generated as part of the Darwin Tree of Life project, which produces reference genomes for eukaryotic species found in Britain and Ireland.

## Species taxonomy

Eukaryota; Viridiplantae; Streptophyta; Streptophytina; Embryophyta; Tracheophyta; Euphyllophyta; Spermatophyta; Magnoliopsida; Mesangiospermae; eudicotyledons; Gunneridae; Pentapetalae; Santalales; Viscaceae; *Viscum*; *Viscum album* L. (NCBI:txid3972)

## Background

**Figure 1.**
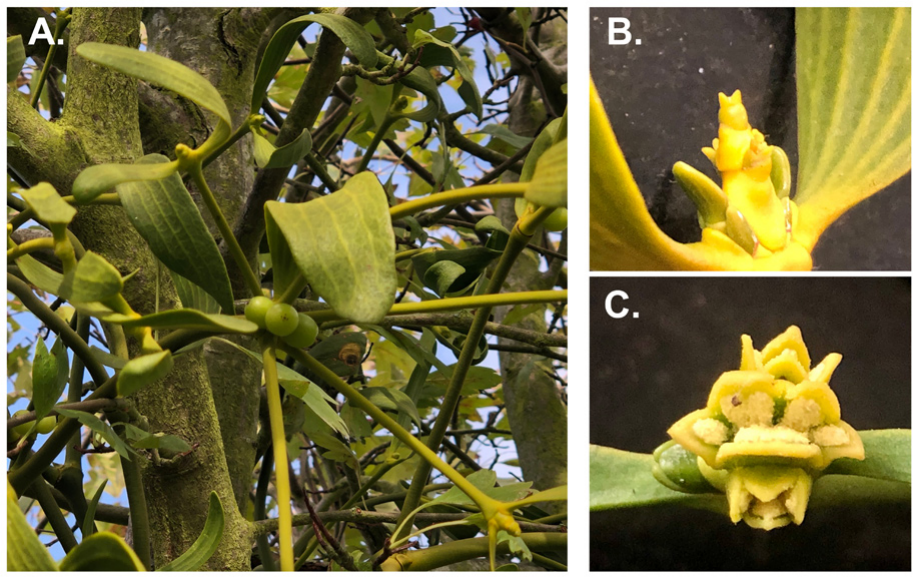
*Viscum album* on hawthorn at Home Park, Hampton Court. ( **A**) The sequenced female individual on its hawthorn host, with developing fruits. ( **B**– **C**) Close-ups of female and male flowers from other individuals in the same population (not the sequenced plant).

The European mistletoe ( *Viscum album*) ( Figure 1) is an evergreen, obligate hemiparasitic shrub that connect via a haustorium to obtain water and inorganic compounds directly from host xylem. They can reach up to 1.50 m across ( Wangerin, 1937). Plants have unisexual scented flowers with four tepals. Fruits are white, globose berries with a single seed ( Thomas *et al.*, 2023).

In Great Britain, mistletoe grows on broadleaf trees, especially poplar ( *Populus*), lime ( *Tilia*), hawthorn ( *Crataegus*), apple trees ( *Malus*) and sometimes on other deciduous species (e.g., *Acer, Aesculus*, *Betula*, *Corylus*, *Juglans*, *Pyrus*, *Quercus*, *Robinia* and *Sorbus*). It is most common in the south and east of Britain, occurring in just a few sites in Scotland although it appears to be expanding there ( Stroh *et al.*, 2023). *Viscum album* is native to most of Europe, except the far north where it is restricted by frost, and in northwest Africa, West Asia and the Himalayas, where its distribution is limited by drought ( Thomas *et al.*, 2023). It has been introduced to Ireland, Japan and California ( Thomas *et al.*, 2023). There are five subspecies, all with different host preferences ( Thomas *et al.*, 2023). In Europe, *Viscum album* subsp. *album* is the only subspecies growing on broadleaf trees, whereas all others prefer gymnosperms. It can be particularly common on trees growing on alkaline soils. Although it does not kill its hosts, the European mistletoe is considered a pest because it reduces fruit yields by up to 50% ( Preston, 1977). Mistletoe branches are often harvested from orchards for sale in Christmas markets.

Because it is evergreen, it is more visible in winter, which likely is the reason for it being a symbol of fertility and vitality in mythology and in folklore of protection, love, winter solstice and Christmas. It was revered by the early herbalists, reputed to cure epileptic trances and tumours, used for divining treasure, keeping witches at bay and protecting crops. It was also reputed to have powers as a fertility potion and aphrodisiac ( Fay *et al.*, 2010) and references therein; ( Christenhusz, 2025; Fay, 2017).

Flowers are pollinated by insects attracted to its fruity scent, such as flies and ants in winter, and later in the spring by bees and bumblebees ( Thomas *et al.*, 2023). Berries of the female plants are an important winter food for a number of bird species, including mistle thrush ( *Turdus viscivorus*) and black caps ( *Sylvia atricapilla*), which are immune to the toxic lectin viscumin in the berries ( Olsnes *et al.*, 1982). These birds disperse the sticky seeds by rubbing them off their beaks or anuses on branches that are suitable for establishment ( Beckman & Sullivan, 2023). The name mistletoe originates from Anglo-Saxon ‘ *mistel*’, dung, and ‘ *tan*’, twig. The sticky fruit pulp resulted in its Latin name, *viscum*, being adopted into English as the word ‘viscous’, sticky. In the past, the berries boiled down to a glue, which was used to catch songbirds for consumption. This resulted in mistletoe being referred to as ‘birdlime’.

Mistletoes have some of the largest genomes among flowering plants: *Viscum* genomes range from 61 to 100 Gbp in size ( Ulrich *et al.*, 1988; Zonneveld, 2010). The *Viscum album* subsp. *album* sampled here has a chromosome-level genome assembly (2 *n* = 20) of 94 Gbp (1C), making it the largest known genome of a plant species native to Britain and Ireland. This work not only represents an enormous technical challenge, but also provides an important genomic resource for the study of the biology of mistletoe, from its genome regulation machinery, its 3-D genome structure, and host-parasite interactions. Although unlikely to provide insights into its mythical powers, this high-quality reference genome is the first step towards the study of the full range of plant genome size and evolutionary consequences of large genomes.

## Methods

### Sample acquisition, flow cytometry and DNA barcoding

The specimen used for genome sequencing was a vegetative structure female *Viscum album* (specimen ID KDTOL10096, ToLID drVisAlbu1), collected from on planted trees along Hampton Court Rd, Home Park, Molesey, Kingston upon Thames, Surrey, United Kingdom (latitude 51.4061, longitude –0.3319) on 2020-09-07. The specimen was collected and identified by Maarten J. M. Christenhusz (Royal Botanic Gardens Kew).

The genome size was estimated by flow cytometry following the ‘one-step’ method outlined in Pellicer *et al*. (2021) and using propidium iodide as the fluorochrome. For this species, was used for isolation of nuclei ( Loureiro *et al.*, 2007), and the internal calibration standard was an *Allium cepa* L. standard as described in Doležel *et al*. (2007).

The initial identification was verified by an additional DNA barcoding process according to the framework developed by Twyford *et al*. (2024). Part of the plant specimen was preserved in silica gel desiccant ( Chase & Hills, 1991). DNA extracted from the dried plant was amplified by PCR for standard barcode markers, with the amplicons sequenced and compared to public sequence databases including GenBank and the Barcode of Life Database (BOLD) ( Ratnasingham & Hebert, 2007). Following whole genome sequence generation, the relevant DNA barcode region was also used alongside the initial barcoding data for sample tracking at the WSI ( Twyford *et al.*, 2024). The standard operating procedures for Darwin Tree of Life barcoding are available on protocols.io.

### Nucleic acid extraction

Protocols for high molecular weight (HMW) DNA extraction developed at the Wellcome Sanger Institute (WSI) Tree of Life Core Laboratory are available on protocols.io ( Howard *et al.*, 2025). The drVisAlbu1 sample was weighed and triaged to determine the appropriate extraction protocol.

Tissue from the leaf was homogenised by cryogenic disruption using the Covaris cryoPREP ^®^ Automated Dry Pulverizer.

HMW DNA was extracted using the Manual Plant MagAttract v2 protocol. DNA was sheared into an average fragment size of 12–20 kb following the Megaruptor®3 for LI PacBio protocol. Sheared DNA was purified by manual SPRI (solid-phase reversible immobilisation). The concentration of the sheared and purified DNA was assessed using a Nanodrop spectrophotometer and Qubit Fluorometer using the Qubit dsDNA High Sensitivity Assay kit. Fragment size distribution was evaluated by running the sample on the FemtoPulse system.

RNA was extracted from flower tissue of drVisAlbu21 in the Tree of Life Laboratory at the WSI using the RNA Extraction: Automated MagMax™ mirVana protocol. The RNA concentration was assessed using a Nanodrop spectrophotometer and a Qubit Fluorometer using the Qubit RNA Broad-Range Assay kit. Analysis of the integrity of the RNA was done using the Agilent RNA 6000 Pico Kit and Eukaryotic Total RNA assay.

### PacBio HiFi library preparation and sequencing

Library preparation and sequencing were performed at the WSI Scientific Operations core. Libraries were prepared using the PacBio Express Template Preparation Kit v2.0 (Pacific Biosciences, California, USA) according to the manufacturer’s instructions. The kit includes the reagents required for removal of single-strand overhangs, DNA damage repair, end repair/A-tailing, adapter ligation, and nuclease treatment. Library preparation also included a library purification step using AMPure PB beads (Pacific Biosciences) and a size-selection step to remove templates <3 kb using AMPure PB modified SPRI. DNA concentration was quantified using the Qubit Fluorometer v2.0 (Thermo Fisher Scientific) and Qubit HS Assay Kit and the final library fragment size analysis was carried out using the Agilent Femto Pulse Automated Pulsed Field CE Instrument (Agilent Technologies).

The sample was sequenced using the Sequel IIe system (Pacific Biosciences, California, USA). The concentration of the library loaded onto the Sequel IIe was in the range 40–135 pM. The SMRT link software, a PacBio web-based end-to-end workflow manager, was used to set-up and monitor the run, and to perform primary and secondary analysis of the data upon completion.

### Hi-C

***Sample preparation and crosslinking***

Hi-C data were generated from frozen leaf tissue of the drVisAlbu1 using the Arima-HiC v2 kit (Arima Genomics). Tissue was finely ground using the Covaris cryoPREP Dry Pulverizer (Covaris), then subjected to nuclei isolation. Nuclei were isolated using a modified protocol based on the Qiagen QProteome Cell Compartment Kit (Qiagen), in which only the Lysis and CE2 buffers were used, with QIAshredder spin columns. After isolation, nuclei were fixed using formaldehyde to a final concentration of 2% to crosslink the DNA. The crosslinked DNA was then digested and biotinylated according to the manufacturer’s instructions. A clean-up step was performed with SPRIselect beads before library preparation. DNA concentration was quantified using the Qubit Fluorometer v4.0 (Thermo Fisher Scientific) and the Qubit HS Assay Kit, following the manufacturer’s instructions.

***Hi-C library preparation and sequencing***

Biotinylated DNA constructs were fragmented using a Covaris E220 sonicator and size selected to 400–600 bp using SPRISelect beads. DNA was enriched with Arima-HiC v2 kit Enrichment beads. End repair, A-tailing, and adapter ligation were carried out with the NEBNext Ultra II DNA Library Prep Kit (New England Biolabs), following a modified protocol where library preparation occurs while DNA remains bound to the Enrichment beads. Library amplification was performed using KAPA HiFi HotStart mix and a custom Unique Dual Index (UDI) barcode set (Integrated DNA Technologies). Depending on sample concentration and biotinylation percentage determined at the crosslinking stage, libraries were amplified with 10–16 PCR cycles. Post-PCR clean-up was performed with SPRISelect beads. Libraries were quantified using the AccuClear Ultra High Sensitivity dsDNA Standards Assay Kit (Biotium) and a FLUOstar Omega plate reader (BMG Labtech).

Prior to sequencing, libraries were normalised to 10 ng/μL. Normalised libraries were quantified again and equimolar and/or weighted 2.8 nM pools. Pool concentrations were checked using the Agilent 4200 TapeStation (Agilent) with High Sensitivity D500 reagents before sequencing. Sequencing was performed using paired-end 150 bp reads on the Illumina NovaSeq 6000.

### RNA library preparation and sequencing

Libraries were prepared using the NEBNext ^®^ Ultra™ II Directional RNA Library Prep Kit for Illumina (New England Biolabs), following the manufacturer’s instructions. Poly(A) mRNA in the total RNA solution was isolated using oligo(dT) beads, converted to cDNA, and uniquely indexed; 14 PCR cycles were performed. Libraries were size-selected to produce fragments between 100–300 bp. Libraries were quantified, normalised, pooled to a final concentration of 2.8 nM, and diluted to 150 pM for loading. Sequencing was carried out on the Illumina NovaSeq X to generate 150-bp paired-end reads.

### Genome assembly

Prior to assembly of the PacBio HiFi reads, a database of *k*-mer counts ( *k* = 31) was generated from the filtered reads using FastK. GenomeScope2 ( Ranallo-Benavidez *et al.*, 2020) was used to analyse the *k*-mer frequency distributions, providing estimates of genome size, heterozygosity, and repeat content.

The HiFi reads were assembled using Hifiasm ( Cheng *et al.*, 2021) with the --primary option. Haplotypic duplications were identified and removed using purge_dups ( Guan *et al.*, 2020). The Hi-C reads ( Rao *et al.*, 2014) were mapped to the primary contigs using bwa-mem2 ( Vasimuddin *et al.*, 2019), and the contigs were scaffolded in YaHS ( Zhou *et al.*, 2023) with the --break option for handling potential misassemblies. The scaffolded assemblies were evaluated using Gfastats ( Formenti *et al.*, 2022), BUSCO ( Manni *et al.*, 2021) and MERQURY.FK ( Rhie *et al.*, 2020).

### Assembly curation

The assembly was decontaminated using the Assembly Screen for Cobionts and Contaminants ( ASCC) pipeline. TreeVal was used to generate the flat files and maps for use in curation. Manual curation was conducted primarily in PretextView and HiGlass ( Kerpedjiev *et al.*, 2018). Scaffolds were visually inspected and corrected as described by Howe *et al*. (2021). Manual corrections included 365 breaks, 353 joins, and removal of 500 haplotypic duplications. The curation process is documented at https://gitlab.com/wtsi-grit/rapid-curation. PretextSnapshot was used to generate a Hi-C contact map of the final assembly.

### Assembly quality assessment

The Merqury.FK tool ( Rhie *et al.*, 2020) was run in a Singularity container ( Kurtzer *et al.*, 2017) to evaluate *k*-mer completeness and assembly quality for the primary and alternate haplotypes using the *k*-mer databases ( *k* = 31) computed prior to genome assembly. The analysis outputs included assembly QV scores and completeness statistics.

The genome was analysed using the BlobToolKit pipeline, a Nextflow implementation of the earlier Snakemake version ( Challis *et al.*, 2020). The pipeline aligns PacBio reads using minimap2 ( Li, 2018) and SAMtools ( Danecek *et al.*, 2021) to generate coverage tracks. It runs BUSCO ( Manni *et al.*, 2021) using lineages identified from NCBI Taxonomy ( Schoch *et al.*, 2020). For the three domain-level lineages, BUSCO genes are aligned to the UniProt Reference Proteomes database ( Bateman *et al.*, 2023) using DIAMOND blastp ( Buchfink *et al.*, 2021).The genome is divided into chunks based on the density of BUSCO genes from the closest taxonomic lineage, and each chunk is aligned to the UniProt Reference Proteomes database with DIAMOND blastx. Sequences without hits are chunked using seqtk and aligned to the NT database with blastn ( Altschul *et al.*, 1990). The BlobToolKit suite consolidates all outputs into a blobdir for visualisation. The BlobToolKit pipeline was developed using nf-core tooling ( Ewels *et al.*, 2020) and MultiQC ( Ewels *et al.*, 2016), with package management via Conda and Bioconda ( Grüning *et al.*, 2018), and containerisation through Docker ( Merkel, 2014) and Singularity ( Kurtzer *et al.*, 2017).

## Genome sequence report

### Sequence data

**Figure 2.**
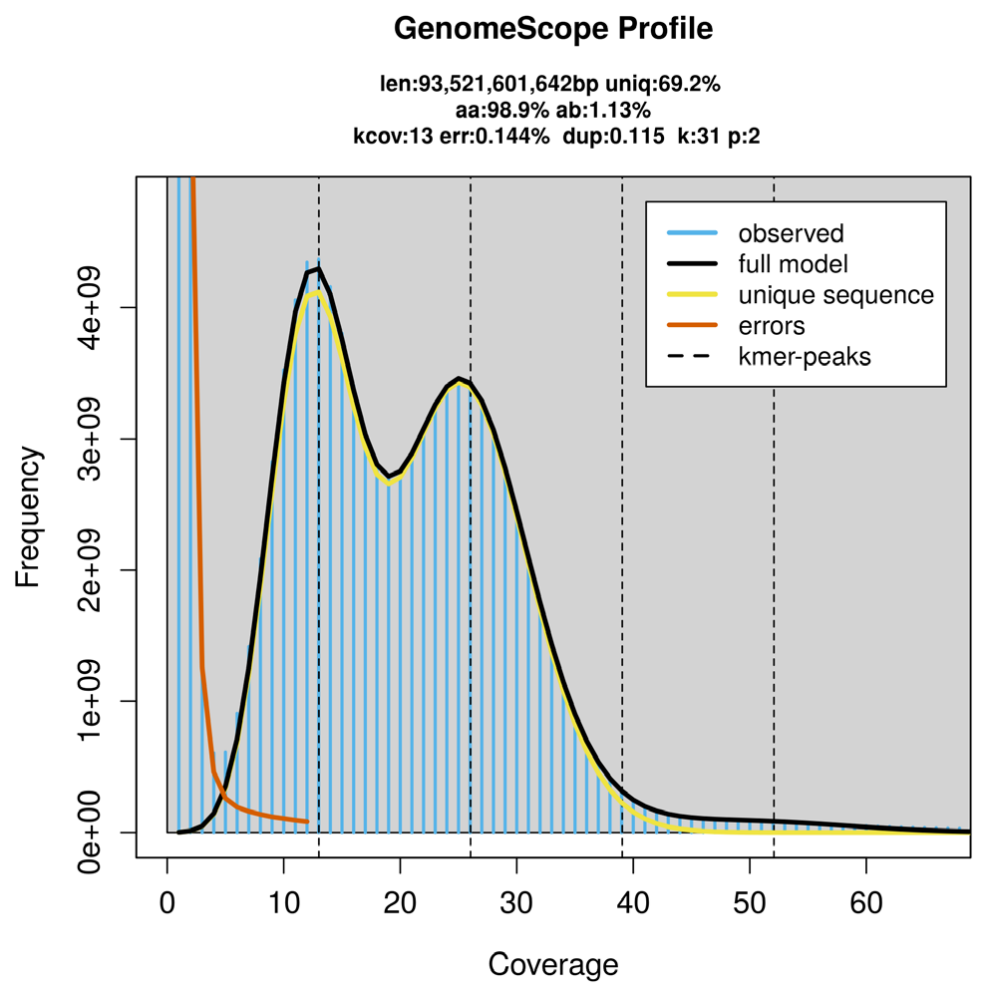
Frequency distribution of *k*-mers generated using GenomeScope2. The plot shows observed and modelled *k*-mer spectra, providing estimates of genome size, heterozygosity, and repeat content based on unassembled sequencing reads.

The genome of a specimen of *Viscum album* was sequenced using Pacific Biosciences single-molecule HiFi long reads, generating 6 011.18 Gb (gigabases) from 393.33 million reads, which were used to assemble the genome. GenomeScope2.0 analysis estimated the haploid genome size at 93 521.60 Mb, with a heterozygosity of 1.13% and repeat content of 30.90% ( Figure 2). Using flow cytometry, the genome size (1C‐value) of the sampled was estimated to be 93.78 pg, equivalent to 91 710.00 Mb. These estimates guided expectations for the assembly. Based on the estimated genome size, the sequencing data provided approximately 26× coverage. Hi-C sequencing produced 1 942.64 Gb from 12 865.20 million reads, which were used to scaffold the assembly. RNA sequencing data were also generated and are available in public sequence repositories. Table 1 summarises the specimen and sequencing details.

**Table 1.** Specimen and sequencing data for BioProject PRJEB47325.

Platform	PacBio HiFi	Hi-C	RNA-seq
**ToLID**	drVisAlbu1	drVisAlbu1	drVisAlbu21
**Specimen ID**	KDTOL10096	KDTOL10096	KDTOL11072
**BioSample (source** **individual)**	SAMEA7522516	SAMEA7522516	SAMEA114565187
**BioSample (tissue)**	SAMEA7522568	SAMEA7522562	SAMEA114565203
**Tissue**	leaf	leaf	flower
**Sequencing platform** **and model**	Sequel IIe	Illumina NovaSeq 6000	Illumina NovaSeq X
**Run accessions**	ERR6808049– ERR8755914	ERR6688777; ERR7220437; ERR7220436	ERR13148236; ERR13148237; ERR11641090; ERR13148235
**Read count total**	393.33 million	12 865.20 million	213.10 million
**Base count total**	6 011.18 Gb	1 942.64 Gb	32.18 Gb

### Assembly statistics

The primary haplotype was assembled, and contigs corresponding to an alternate haplotype were also deposited in INSDC databases. The final assembly has a total length of 94 261.04 Mb in 5 096 scaffolds, with 6 368 gaps, and a scaffold N50 of 2 134.69 Mb ( Table 2).

**Table 2.** Genome assembly statistics.

**Assembly name**	drVisAlbu1.1
**Assembly accession**	GCA_963277665.1
**Alternate haplotype accession**	GCA_963082535.1
**Assembly level**	chromosome
**Span (Mb)**	94 261.04
**Number of chromosomes**	11
**Number of contigs**	11 464
**Contig N50**	34.83 Mb
**Number of scaffolds**	5 096
**Scaffold N50**	2134.69 Mb
**Supernumerary chromosomes**	B _1_

**Figure 3.**
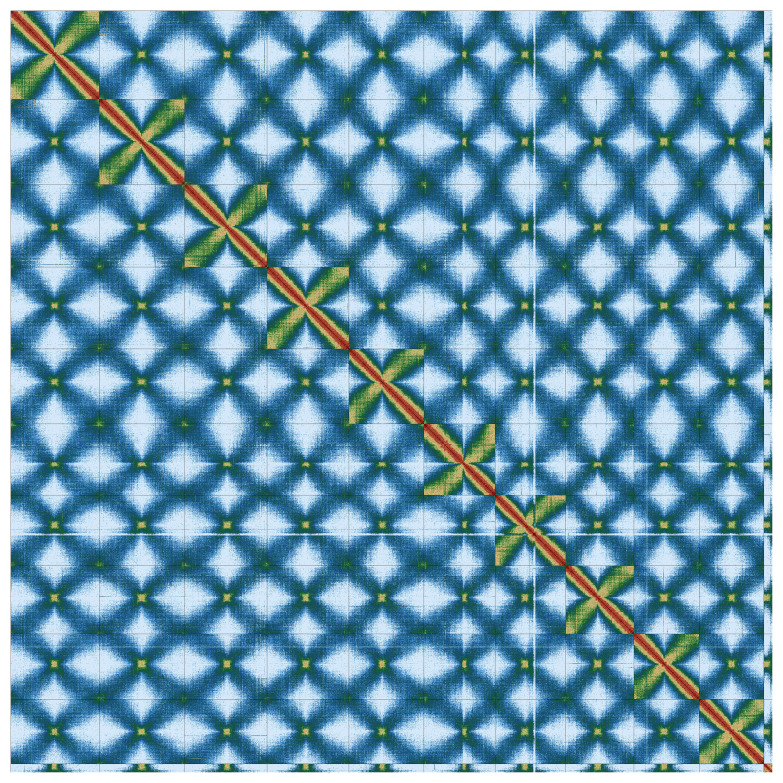
Hi-C contact map of the *Viscum album* genome assembly. Assembled chromosomes are shown in order of size. The plot was generated using PretextSnapshot.

Most of the assembly sequence (98.67%) was assigned to 11 chromosomal-level scaffolds, representing 10 autosomes and the B _1_ chromosome. These chromosome-level scaffolds, confirmed by Hi-C data, are named according to size ( Figure 3; Table 3). Because several chromosome sequences exceed the INSDC per-record length limit (2,147,483,647 bp), they were deposited in consecutive parts with separate accessions; in total, the 11 chromosomes correspond to 49 accessions.

**Table 3.** Chromosomal pseudomolecules in the primary genome assembly of *Viscum album* drVisAlbu1. The 11 chromosomes were submitted to the INSDC in multiple parts due to length constraints.

INSDC accession	Molecule	Length (Mb)	GC%
OY728119.1	1_1	2 143.53	31.50
OY728120.1	1_2	2 138.63	31
OY728121.1	1_3	2 132.99	31
OY728122.1	1_4	2 142.15	31
OY728123.1	1_5	2 142.78	31.50
OY728124.1	1_6	124.38	32.50
OY728125.1	2_1	2 112.40	31.50
OY728126.1	2_2	2 144.48	31
OY728127.1	2_3	2 133.12	31
OY728128.1	2_4	2 141.81	31
OY728129.1	2_5	1 870.27	31.50
OY728130.1	3_1	2 134.93	31.50
OY728131.1	3_2	2 108.66	31
OY728132.1	3_3	2 146.28	31
OY728133.1	3_4	2 117.02	31
OY728134.1	3_5	1 576.30	31.50
OY728135.1	4_1	2 067.10	31.50
OY728136.1	4_2	2 134.69	31
OY728137.1	4_3	2 136.66	31
OY728138.1	4_4	2 140.54	31
OY728139.1	4_5	1 531.58	31.50
OY728140.1	5_1	2 146.57	31.50
OY728141.1	5_2	2 138.19	31
OY728142.1	5_3	2 101.18	31
OY728143.1	5_4	2 146.23	31.50
OY728144.1	5_5	621.09	32
OY728145.1	6_1	2 138.61	31.50
OY728146.1	6_2	2 083.69	31
OY728147.1	6_3	2 144.31	31
OY728148.1	6_4	2 139.18	31
OY728149.1	6_5	172.72	32
OY728150.1	7_1	2 132.15	31
OY728151.1	7_2	2 133.92	31
OY728152.1	7_3	2 133.31	31
OY728153.1	7_4	2 100.93	31.50
OY728154.1	7_5	143.35	32
OY728155.1	8_1	2 134.14	31.50
OY728156.1	8_2	2 145.20	31
OY728157.1	8_3	2 137.73	31
OY728158.1	8_4	1 914.31	31.50
OY728159.1	9_1	2 145.48	31
OY728160.1	9_2	2 114.17	31
OY728161.1	9_3	2 146.42	31
OY728162.1	9_4	1 555.47	31.50
OY728163.1	10_1	2 141.25	31.50
OY728164.1	10_2	2 119.19	31
OY728165.1	10_3	2 142.18	31
OY728166.1	10_4	1 498.83	31.50
OY728167.1	B _1_	1 020.19	32

### Assembly quality metrics

**Figure 4.**
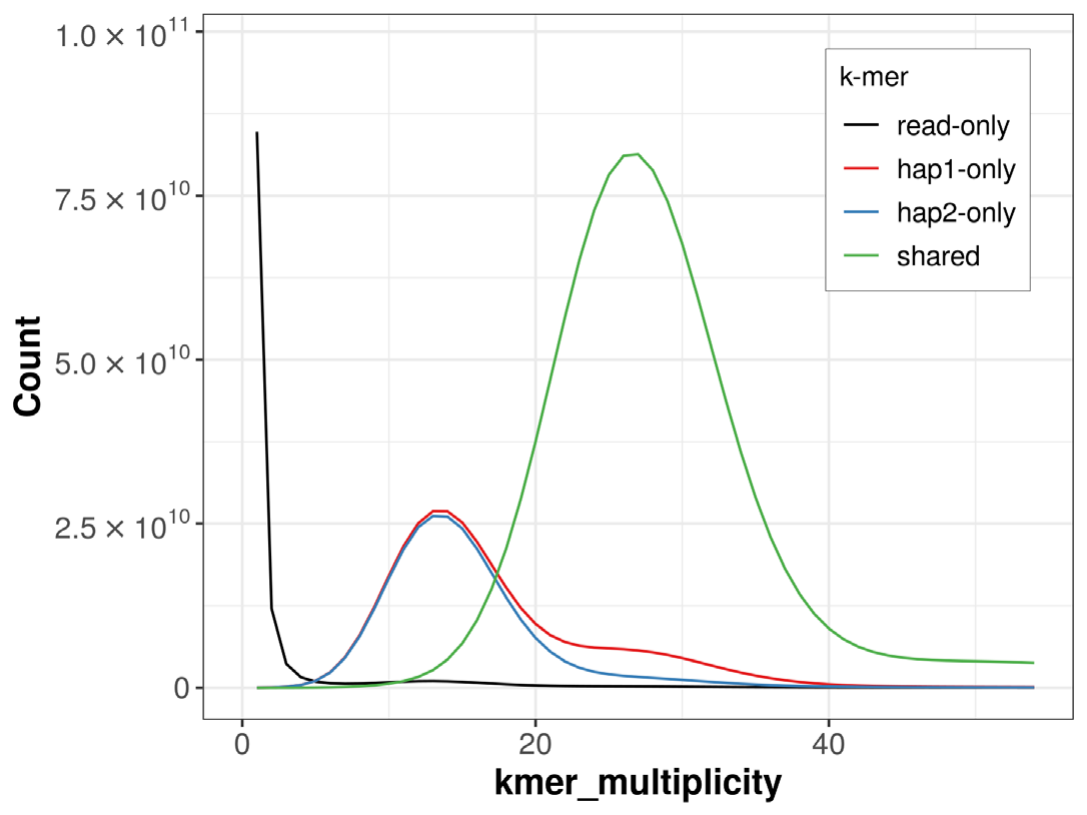
Evaluation of *k*-mer completeness using MerquryFK. This plot illustrates the recovery of *k*-mers from the original read data in the final assemblies. The horizontal axis represents *k*-mer multiplicity, and the vertical axis shows the number of *k*-mers. The black curve represents *k*-mers that appear in the reads but are not assembled. The green curve (the homozygous peak) corresponds to *k*-mers shared by both haplotypes and the red and blue curves (the heterozygous peaks) show *k*-mers found only in one of the haplotypes.

**Figure 5.**
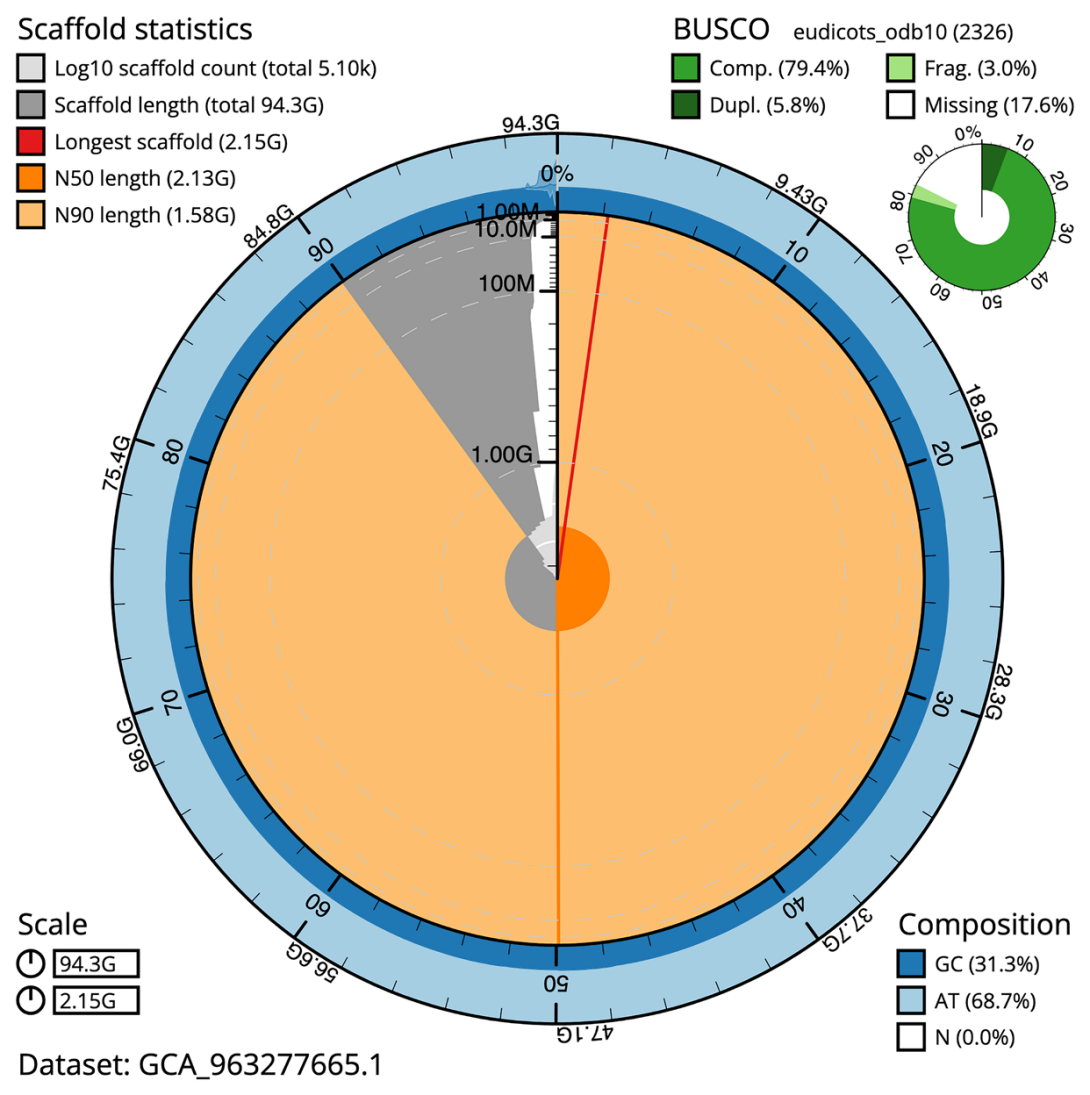
Assembly metrics for drVisAlbu1.1. The BlobToolKit snail plot provides an overview of assembly metrics and BUSCO gene completeness. The circumference represents the length of the whole genome sequence, and the main plot is divided into 1,000 bins around the circumference. The outermost blue tracks display the distribution of GC, AT, and N percentages across the bins. Scaffolds are arranged clockwise from longest to shortest and are depicted in dark grey. The longest scaffold is indicated by the red arc, and the deeper orange and pale orange arcs represent the N50 and N90 lengths. A light grey spiral at the centre shows the cumulative scaffold count on a logarithmic scale. A summary of complete, fragmented, duplicated, and missing BUSCO genes in the eudicots_odb10 set is presented at the top right. An interactive version of this figure can be accessed on the BlobToolKit viewer.

**Figure 6.**
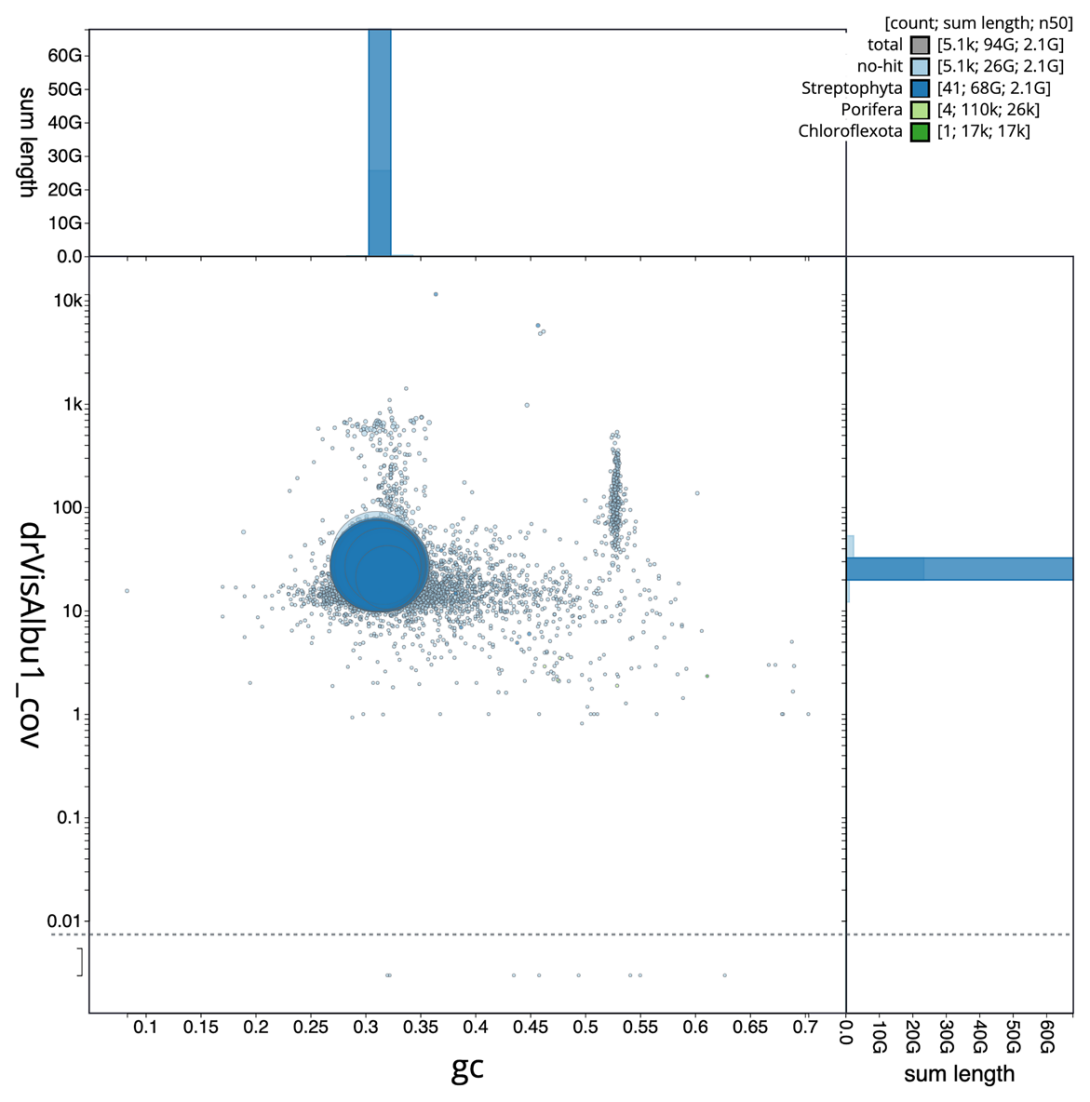
BlobToolKit GC-coverage plot for drVisAlbu1.1. Blob plot showing sequence coverage (vertical axis) and GC content (horizontal axis). The circles represent scaffolds, with the size proportional to scaffold length and the colour representing phylum membership. The histograms along the axes display the total length of sequences distributed across different levels of coverage and GC content. An interactive version of this figure is available on the BlobToolKit viewer.

The combined primary and alternate assemblies achieve an estimated QV of 67.3. The *k*-mer completeness is 76.68% for the primary assembly, 73.77% for the alternate haplotype, and 98.59% for the combined assemblies ( Figure 4). BUSCO analysis using the eudicots_odb10 reference set ( *n* = 2 326) identified 79.4% of the expected gene set (single = 73.7%, duplicated = 5.8%). The snail plot in Figure 5 summarises the scaffold length distribution and other assembly statistics for the primary assembly. The blob plot in Figure 6 shows the distribution of scaffolds by GC proportion and coverage.

Table 4 lists the assembly metric benchmarks adapted from Rhie *et al.* (2021) the Earth BioGenome Project Report on Assembly Standards September 2024. The EBP metric calculated for the primary assembly is **7.C.Q67**, meeting the recommended reference standard.

**Table 4.** Earth Biogenome Project summary metrics for the *Viscum album* assembly.

Measure	Value	Benchmark
EBP summary (primary)	7.C.Q67	6.C.Q40
Contig N50 length	34.83 Mb	≥ 1 Mb
Scaffold N50 length	2 134.69 Mb	= chromosome N50
Consensus quality (QV)	Primary: 67.3; alternate: 67.2; combined: 67.3	≥ 40
*k*-mer completeness	Primary: 76.68%; alternate: 73.77%; combined: 98.59%	≥ 95%
BUSCO	C:79.4%[S:73.7%;D:5.8%]; F:3.0%; M:17.6%; n:2 326	S > 90%; D < 5%
Percentage of assembly assigned to chromosomes	98.67%	≥ 90%

### Wellcome Sanger Institute – Legal and Governance

The materials that have contributed to this genome note have been supplied by a Darwin Tree of Life Partner. The submission of materials by a Darwin Tree of Life Partner is subject to the **‘Darwin Tree of Life Project Sampling Code of Practice’**, which can be found in full on the Darwin Tree of Life website. By agreeing with and signing up to the Sampling Code of Practice, the Darwin Tree of Life Partner agrees they will meet the legal and ethical requirements and standards set out within this document in respect of all samples acquired for, and supplied to, the Darwin Tree of Life Project. Further, the Wellcome Sanger Institute employs a process whereby due diligence is carried out proportionate to the nature of the materials themselves, and the circumstances under which they have been/are to be collected and provided for use. The purpose of this is to address and mitigate any potential legal and/or ethical implications of receipt and use of the materials as part of the research project, and to ensure that in doing so we align with best practice wherever possible. The overarching areas of consideration are:

- Ethical review of provenance and sourcing of the material
- Legality of collection, transfer and use (national and international)

Each transfer of samples is further undertaken according to a Research Collaboration Agreement or Material Transfer Agreement entered into by the Darwin Tree of Life Partner, Genome Research Limited (operating as the Wellcome Sanger Institute), and in some circumstances, other Darwin Tree of Life collaborators.

## Data Availability

European Nucleotide Archive: Viscum album (European mistletoe). Accession number PRJEB47325. The genome sequence is released openly for reuse. The *Viscum album* genome sequencing initiative is part of the Darwin Tree of Life Project (PRJEB40665) and the Sanger Institute Tree of Life Programme (PRJEB43745). All raw sequence data and the assembly have been deposited in INSDC databases. The genome will be annotated using available RNA-Seq data and presented through the Ensembl pipeline at the European Bioinformatics Institute. Raw data and assembly accession identifiers are reported in Table 1 and Table 2. Pipelines used for genome assembly at the WSI Tree of Life are available at https://pipelines.tol.sanger.ac.uk/pipelines. Table 5 lists software versions used in this study.

## Author information

- Members of the Royal Botanic Gardens Kew Genome Acquisition Lab
- Members of the Royal Botanic Garden Edinburgh Genome Acquisition Lab
- Members of the Plant Genome Sizing collective
- Members of the Wellcome Sanger Institute Tree of Life Management, Samples and Laboratory team
- Members of Wellcome Sanger Institute Scientific Operations – Sequencing Operations
- Members of the Wellcome Sanger Institute Tree of Life Core Informatics team
- Members of the Tree of Life Core Informatics collective
- Members of the Darwin Tree of Life Consortium

## References

[ref-1] Altschul SF Gish W Miller W : Basic Local Alignment Search Tool. *J Mol Biol.* 1990;215(3):403–410. 10.1016/S0022-2836(05)80360-2 2231712

[ref-2] Bateman A Martin MJ Orchard S : UniProt: the Universal Protein Knowledgebase in 2023. *Nucleic Acids Res.* 2023;51(D1):D523–D531. 10.1093/nar/gkac1052 36408920 PMC9825514

[ref-3] Beckman NG Sullivan LL : The causes and consequences of seed dispersal. *Annu Rev Ecol Evol Syst.* 2023;54(1):403–27. 10.1146/annurev-ecolsys-102320-104739

[ref-4] Buchfink B Reuter K Drost HG : Sensitive protein alignments at Tree-of-Life scale using DIAMOND. *Nat Methods.* 2021;18(4):366–368. 10.1038/s41592-021-01101-x 33828273 PMC8026399

[ref-5] Challis R Richards E Rajan J : BlobToolKit – interactive quality assessment of genome assemblies. *G3 (Bethesda).* 2020;10(4):1361–1374. 10.1534/g3.119.400908 32071071 PMC7144090

[ref-6] Chase MW Hills HH : Silica gel: an ideal material for field preservation of leaf samples for DNA studies. *Taxon.* 1991;40(2):215–20. 10.2307/1222975

[ref-7] Cheng H Concepcion GT Feng X : Haplotype-resolved *de novo* assembly using phased assembly graphs with hifiasm. *Nat Methods.* 2021;18(2):170–175. 10.1038/s41592-020-01056-5 33526886 PMC7961889

[ref-8] Christenhusz M : Liefde in de plantenwereld, een botanische kijk op de evolutie van seks. Sterck & De Vreese,2025. Reference Source

[ref-9] Danecek P Bonfield JK Liddle J : Twelve years of SAMtools and BCFtools. *GigaScience.* 2021;10(2): giab008. 10.1093/gigascience/giab008 33590861 PMC7931819

[ref-10] Doležel J Greilhuber J Suda J : Estimation of nuclear DNA content in plants using flow cytometry. *Nat Protoc.* 2007;2(9):2233–44. 10.1038/nprot.2007.310 17853881

[ref-11] Ewels P Magnusson M Lundin S : MultiQC: summarize analysis results for multiple tools and samples in a single report. *Bioinformatics.* 2016;32(19):3047–3048. 10.1093/bioinformatics/btw354 27312411 PMC5039924

[ref-12] Ewels PA Peltzer A Fillinger S : The nf-core framework for community-curated bioinformatics pipelines. *Nat Biotechnol.* 2020;38(3):276–278. 10.1038/s41587-020-0439-x 32055031

[ref-13] Fay MF : Is mistletoe more than just an excuse for a kiss? 2017. Reference Source

[ref-14] Fay MF Bennett JR Dixon KW : Parasites, their relationships and the disintegration of scrophulariaceae *Sensu lato*. *Curtis’s Botanical Magazine.* 2010;26(4):286–313. 10.1111/j.1467-8748.2009.01668.x

[ref-15] Formenti G Abueg L Brajuka A : Gfastats: conversion, evaluation and manipulation of genome sequences using assembly graphs. *Bioinformatics.* 2022;38(17):4214–4216. 10.1093/bioinformatics/btac460 35799367 PMC9438950

[ref-16] Grüning B Dale R Sjödin A : Bioconda: sustainable and comprehensive software distribution for the life sciences. *Nat Methods.* 2018;15(7):475–476. 10.1038/s41592-018-0046-7 29967506 PMC11070151

[ref-17] Guan D McCarthy SA Wood J : Identifying and removing haplotypic duplication in primary genome assemblies. *Bioinformatics.* 2020;36(9):2896–2898. 10.1093/bioinformatics/btaa025 31971576 PMC7203741

[ref-18] Howard C Denton A Jackson B : On the path to reference genomes for all biodiversity: lessons learned and laboratory protocols created in the Sanger Tree of Life core laboratory over the first 2000 species. *bioRxiv.* 2025. 10.1101/2025.04.11.648334 PMC12548527 41129326

[ref-19] Howe K Chow W Collins J : Significantly improving the quality of genome assemblies through curation. *GigaScience.* 2021;10(1): giaa153. 10.1093/gigascience/giaa153 33420778 PMC7794651

[ref-20] Kerpedjiev P Abdennur N Lekschas F : HiGlass: web-based visual exploration and analysis of genome interaction maps. *Genome Biol.* 2018;19(1): 125. 10.1186/s13059-018-1486-1 30143029 PMC6109259

[ref-21] Kurtzer GM Sochat V Bauer MW : Singularity: scientific containers for mobility of compute. *PLoS One.* 2017;12(5): e0177459. 10.1371/journal.pone.0177459 28494014 PMC5426675

[ref-22] Li H : Minimap2: pairwise alignment for nucleotide sequences. *Bioinformatics.* 2018;34(18):3094–3100. 10.1093/bioinformatics/bty191 29750242 PMC6137996

[ref-23] Loureiro J Rodriguez E Doležel J : Two new nuclear isolation buffers for plant DNA flow cytometry: a test with 37 species. *Ann Bot.* 2007;100(4):875–88. 10.1093/aob/mcm152 17684025 PMC2749623

[ref-24] Manni M Berkeley MR Seppey M : BUSCO update: novel and streamlined workflows along with broader and deeper phylogenetic coverage for scoring of eukaryotic, prokaryotic, and viral genomes. *Mol Biol Evol.* 2021;38(10):4647–4654. 10.1093/molbev/msab199 34320186 PMC8476166

[ref-25] Merkel D : Docker: lightweight Linux containers for consistent development and deployment. *Linux J.* 2014;2014(239): 2. Reference Source

[ref-26] Olsnes S Stirpe F Sandvig K : Isolation and characterization of viscumin, a toxic lectin from *Viscum album* L. (mistletoe). *J Biol Chem.* 1982;257(22):13263–70. 10.1016/S0021-9258(18)33440-9 7142144

[ref-27] Pellicer J Powell RF Leitch IJ : The application of flow cytometry for estimating genome size, ploidy level endopolyploidy, and reproductive modes in plants.In: P. Besse (ed), *Methods Mol Biol.* New York, NY,2021;2222:325–61. 10.1007/978-1-0716-0997-2_17 33301101

[ref-28] Preston AP : Effects of mistletoe ( *Viscum album*) on young apple trees. *Hortic Res.* 1977;17:33–38.

[ref-29] Ranallo-Benavidez TR Jaron KS Schatz MC : GenomeScope 2.0 and Smudgeplot for reference-free profiling of polyploid genomes. *Nat Commun.* 2020;11(1): 1432. 10.1038/s41467-020-14998-3 32188846 PMC7080791

[ref-30] Rao SSP Huntley MH Durand NC : A 3D map of the human genome at kilobase resolution reveals principles of chromatin looping. *Cell.* 2014;159(7):1665–1680. 10.1016/j.cell.2014.11.021 25497547 PMC5635824

[ref-31] Ratnasingham S Hebert PDN : BOLD: the Barcode of Life Data system ( http://www.barcodinglife.org). *Mol Ecol Notes.* 2007;7(3):355–64. 10.1111/j.1471-8286.2007.01678.x 18784790 PMC1890991

[ref-32] Rhie A McCarthy SA Fedrigo O : Towards complete and error-free genome assemblies of all vertebrate species. *Nature.* 2021;592(7856):737–746. 10.1038/s41586-021-03451-0 33911273 PMC8081667

[ref-33] Rhie A Walenz BP Koren S : Merqury: reference-free quality, completeness, and phasing assessment for genome assemblies. *Genome Biol.* 2020;21(1): 245. 10.1186/s13059-020-02134-9 32928274 PMC7488777

[ref-34] Schoch CL Ciufo S Domrachev M : NCBI taxonomy: a comprehensive update on curation, resources and tools. *Database (Oxford).* 2020;2020: baaa062. 10.1093/database/baaa062 32761142 PMC7408187

[ref-35] Stroh PA Walker KJ Humphrey TA : Plant Atlas 2020: mapping changes in the distribution of the British and Irish flora. Princeton University Press,2023. 10.2307/j.ctv2x6f08m

[ref-36] Thomas PA Dering M Giertych MJ : Biological flora of Britain and Ireland: *Viscum album*. *J Ecol.* 2023;111(3):701–39. 10.1111/1365-2745.14036

[ref-37] Twyford AD Beasley J Barnes I : A DNA barcoding framework for taxonomic verification in the Darwin Tree of Life project [version 1; peer review: 2 approved]. *Wellcome Open Res.* 2024;9:339. 10.12688/wellcomeopenres.21143.1 39386966 PMC11462125

[ref-38] Ulrich I Fritz B Ulrich W : Application of DNA fluorochromes for flow cytometric DNA analysis of plant protoplasts. *Plant Sci.* 1988;55(2):151–58. 10.1016/0168-9452(88)90171-9

[ref-39] Vasimuddin M Misra S Li H : Efficient architecture-aware acceleration of BWA-MEM for multicore systems.In: *2019 IEEE International Parallel and Distributed Processing Symposium (IPDPS).*IEEE,2019;314–324. 10.1109/IPDPS.2019.00041

[ref-40] Wangerin W : Loranthaceae.In: O. v. Kirchner, E. Loew, and C. Schroeter (eds), *Lebensgeschichte der Blütenpflanzen Mitteleuropas.*Stuttgart: Ulmer,1937;II/1:953–1146. Reference Source

[ref-41] Zhou C McCarthy SA Durbin R : YaHS: Yet another Hi-C Scaffolding tool. *Bioinformatics.* 2023;39(1): btac808. 10.1093/bioinformatics/btac808 36525368 PMC9848053

[ref-42] Zonneveld BJM : New record holders for maximum genome size in eudicots and monocots. *J Bot.* 2010;2010(1): 527357. 10.1155/2010/527357

