# Supplementary material for: The genome sequence of the European mistletoe, Viscum album L. (Santalales: Viscaceae)
Source: Wellcome Open Res. 2025 Sep 15;10:517. [Version 1] doi: 10.12688/wellcomeopenres.24920.1 (PMC13058578; doi:10.12688/wellcomeopenres.24920.1)
Supplement: Peer review history

# Reviewer report: Reviewer response for version 1

**DOI:** 10.21956/wellcomeopenres.27450.r151072

**Published:** 2026-04-23

**Authors:** Sunil Kumar Sahu

**Affiliations:** 1 Key Laboratory of Genomics, Ministry of Agriculture, BGI Research, Shenzhen, China

This Data Note provides clear value by presenting a chromosome-scale nuclear genome for *Viscum album*, a species notable for its unusually large genome and biological significance as an obligate hemiparasite. The assembly is technically impressive and fills an important gap in mistletoe genomics, particularly for studies of parasitism, genome expansion, and chromosome-scale organization.

 That said, the manuscript would benefit from more explicitly positioning this advance in relation to prior *V. album* genomic resources, including previously published gene-space and organelle-focused studies referenced in the review materials.

1. The sequencing and assembly strategy is broadly appropriate for a large and complex plant genome, incorporating PacBio HiFi, Hi-C, RNA-seq, Hifiasm, YaHS, and manual curation. However, several methodological details are not described with sufficient clarity to ensure reproducibility. In particular, the flow cytometry protocol, the DNA barcoding loci used, and the provenance of the RNA-seq samples relative to the genome specimen should be more explicitly documented.
2. The manuscript should also clarify the curation workflow. Specifically, it would be helpful to distinguish which steps were performed using PretextView versus HiGlass, rather than listing both tools without context. As written, the methods are generally sound but not fully reproducible.
3. The assembly statistics are strong, with a chromosome-scale genome, high scaffold N50, and a high QV score. However, there is a notable internal inconsistency in the reported HiFi sequencing coverage. The stated 6,011.18 Gb of HiFi data and an approximately 91.7 Gb genome size are not consistent with the reported 26-fold coverage. This discrepancy should be corrected or clearly explained, including whether the coverage estimate reflects filtered reads only and, if so, the criteria used for filtering.
4. In Figure 3, the Hi-C contact map lacks appropriate labeling. Please update the figure to improve interpretability.
5. In Figure 4, the proportions appear slightly inconsistent: if the unique fraction is 69.2%, the repeat fraction should be 30.8%, not 30.9% as stated in the text. This should be corrected.
6. The phrase “49 chromosomal pseudomolecules” may be misinterpreted as indicating 49 chromosomes. It would be clearer to specify that the assembly represents 11 chromosomes, with sequences split across multiple INSDC accessions due to sequence-length limitations.
7. While the manuscript is generally readable, several presentation issues reduce overall clarity and confidence. These include minor grammatical errors, inconsistent unit formatting, and awkward phrasing (e.g., “collected from on planted trees”), all of which should be corrected through careful language editing.

Are sufficient details of methods and materials provided to allow replication by others?

Partly

Is the rationale for creating the dataset(s) clearly described?

Yes

Are the datasets clearly presented in a useable and accessible format?

Yes

Are the protocols appropriate and is the work technically sound?

Yes

Reviewer Expertise:

Plant genomics and evolution

I confirm that I have read this submission and believe that I have an appropriate level of expertise to confirm that it is of an acceptable scientific standard, however I have significant reservations, as outlined above.

# Reviewer report: Reviewer response for version 1

**DOI:** 10.21956/wellcomeopenres.27450.r147744

**Published:** 2026-04-07

**Authors:** Rizky Dwi Satrio

**Affiliations:** 1 The Republic of Indonesia Defense University, Bogor, Indonesia

This manuscript presents a chromosome-level genome assembly of *Viscum album*, an obligate hemiparasitic plant with an exceptionally large genome (~94 Gb). The study represents a significant technical achievement, and the generation and analysis of genomic data at this scale is clearly a non-trivial effort that deserves strong appreciation, given the substantial computational and methodological challenges involved. The integration of PacBio HiFi sequencing, Hi-C scaffolding, and comprehensive assembly curation is appropriate and aligns with current best practices.

 However, as a Data Note, the manuscript is primarily descriptive and lacks deeper biological interpretation, functional insights, and critical discussion of assembly limitations. While the dataset is valuable, several methodological clarifications, inconsistencies in reporting, and issues related to genome quality metrics and completeness should be addressed to improve clarity, reproducibility, and scientific rigor.

**1. Novelty and Biological Significance**

 The manuscript strongly emphasizes the technical achievement but provides limited biological insight into *Viscum album*. The authors should include a brief discussion on genome evolution in parasitic plants, implications of extremely large genome size (e.g., transposable element expansion and gene family evolution), and comparisons with other large plant genomes.

**2. BUSCO Completeness is Relatively Low**

 The reported BUSCO completeness of 79.4% is below the recommended benchmark (>90%) and raises concerns about incomplete gene space representation. The authors should provide a clear explanation for this result (e.g., genome size, repeat content, or lineage divergence) and consider including additional completeness metrics or lineage-specific BUSCO analyses.

**3. K-mer Completeness Discrepancy**

 The primary assembly shows relatively low k-mer completeness (76.68%) compared to the combined assembly (98.59%), suggesting missing or partitioned genomic information. The authors should clarify whether the primary assembly is intended as the reference and discuss the implications of this discrepancy for downstream analyses.

**4. Limited Discussion of Repeat Content**

 Although repeat content (~30.9%) is reported, it is not further analyzed or discussed in the manuscript. Given the extremely large genome size, the authors should provide a repeat annotation summary (e.g., TE classes and proportions) and compare these findings with other large plant genomes.

**5. Lack of Gene Annotation Results**

 No gene annotation results are presented, and this is only mentioned as future work, which limits the utility of the dataset. The authors should include at least a preliminary gene prediction or clearly justify its omission as a scope limitation of this Data Note.

**6. Coverage is Relatively Low for Genome Size**

 The sequencing depth (~26×) may be relatively low considering the extremely large genome size, potentially affecting assembly completeness and accuracy. The authors should justify the adequacy of this coverage for HiFi-based assembly and discuss possible biases or missing genomic regions.

**7. RNA-seq Sample Inconsistency**

 RNA-seq data were generated from a different individual (drVisAlbu21) than the genome assembly sample (drVisAlbu1), which may introduce inconsistencies for annotation. The authors should justify the use of a different individual and flower tissue, clarify its relevance, and discuss implications for downstream annotation, while ideally providing genome annotation and making it publicly accessible even within the Data Note framework.

**8. Chromosome Number vs Assembly Representation**

 The species is reported as 2n = 20, yet the assembly is described as 10 autosomes plus a B chromosome, which may cause confusion. The authors should clearly explain the haploid versus diploid representation and explicitly describe the chromosome naming and counting scheme.

**9. Overly Descriptive Background Section**

 The background section includes folklore and cultural descriptions that are not directly relevant to the genomic focus of the study. The authors should streamline this section and focus more on genomic, evolutionary, and biological context.

**10. Limited Critical Evaluation of Assembly Quality**

 Although the assembly meets EBP standards (7.C.Q67), the manuscript lacks critical discussion of its limitations. The authors should include a concise limitations paragraph addressing BUSCO incompleteness, potential collapsed repeats, and unresolved heterozygosity.

Are sufficient details of methods and materials provided to allow replication by others?

Yes

Is the rationale for creating the dataset(s) clearly described?

Yes

Are the datasets clearly presented in a useable and accessible format?

Yes

Are the protocols appropriate and is the work technically sound?

Yes

Reviewer Expertise:

My expertise lies in plant genomics and bioinformatics, with a strong focus on genome assembly, annotation, analysis, and interpretation of complex plant genomes.

I confirm that I have read this submission and believe that I have an appropriate level of expertise to confirm that it is of an acceptable scientific standard, however I have significant reservations, as outlined above.

# Reviewer report: Reviewer response for version 1

**DOI:** 10.21956/wellcomeopenres.27450.r151067

**Published:** 2026-03-27

**Authors:** Xin Liu

**Affiliations:** 1 Beijing Genomics Institute, Beijing, China

This Data Note describes the sequencing and assembly of the European mistletoe genome. For a non‑UK reviewer like myself, the authors’ engaging background section provided valuable insight into the cultural (and even medicinal) significance of this species. As a genomics researcher, I was already aware of the unusually large genome size in Viscum species, and it is an honor to review this article, which adds meaningful knowledge to plant genomics.

 The Darwin Tree of Life project has supported numerous genome studies across plants and animals. Publishing a Data Note is an effective way to rapidly benefit the wider research community. I also recognize that a Data Note differs from a research article: the goal is to clearly describe methods, present the data, and summarize the results. Within this framework, I find this Data Note already very strong. I have no major concerns, but I would like to share a few comments and suggestions—these are not scientific criticisms, but rather ideas for clarifying the interpretation of the genomic data:

1. **Genome assembly quality** for a genome exceeding 90 Gb, the assembly achieved here is impressive and beyond my expectations. It is clear that the authors invested substantial effort and computational resources in the analysis. The methods and workflows described will serve as a valuable reference for future large‑genome projects. This is a major contribution, and I thank the authors for advancing plant genomics.
2. **Assembly validation through sequencing depth** Sequence alignment already provides strong evidence of assembly quality. Since the authors have performed alignments, I suggest adding a simple figure showing the distribution of sequencing depth across bases. Such a plot would clearly illustrate accuracy (e.g., whether it resembles a Poisson distribution), coverage, and completeness (mapping rate). I do not usually request this in reviews, but for a genome of this scale, such a straightforward visualization would provide highly informative quality metrics.
3. **Scaffold statistics beyond chromosomes** Although the assembly contains over 5,000 scaffolds, the main chromosomes are relatively complete, as noted in the manuscript. For the smaller scaffolds, I recommend providing basic statistics—such as their length distribution after excluding the chromosome‑level sequences. While deeper biological interpretation may be reserved for a future research article, even preliminary statistics in the Data Note would help readers understand the assembly outcome and infer characteristics of the Viscum genome.
4. **Repeat content from k‑mer analysis** The k‑mer analysis suggests that approximately 30% of the genome consists of repetitive sequences. This is intriguing, and although I expect the authors will explore this further in subsequent work, it would be useful to briefly highlight possible reasons here.

Are sufficient details of methods and materials provided to allow replication by others?

Yes

Is the rationale for creating the dataset(s) clearly described?

Yes

Are the datasets clearly presented in a useable and accessible format?

Yes

Are the protocols appropriate and is the work technically sound?

Yes

Reviewer Expertise:

genomics

I confirm that I have read this submission and believe that I have an appropriate level of expertise to confirm that it is of an acceptable scientific standard.

# Reviewer report: Reviewer response for version 1

**DOI:** 10.21956/wellcomeopenres.27450.r147747

**Published:** 2026-03-10

**Authors:** Juan Francisco Ornelas, Enrique Ibarra-Laclette, Saddan Morales-Saldaña

**Affiliations:** 1 Red de Biología Evolutiva, Instituto de Ecología, A.C. (INECOL), Xalapa, Veracruz, Mexico; 2 Red de Estudios Moleculares Avanzados (REMAv), Instituto de Ecología, A.C. (INECOL, Veracruz, Mexico; 3 Herbario, Universidad Nacional Autonoma de Mexico Facultad de Ciencias (Ringgold ID: 98804), Mexico City, Mexico

**Review of Wellcome Open Research WOR-v1-2026-0517**

 Lucia Campos-Dominguez, Maarten J. M. Christenhusz, David Bell, Michael F. Fay, Michelle Hart, Peter M. Hollingsworth, Ilia J. Leitch, Sahr Mian, Kanae Nishii, Royal Botanic Gardens Kew, Royal Botanic Garden Edinburgh, Darwin Tree of Life Barcoding Collective, Wellcome Sanger Institute Tree of Life Management, Samples and Laboratory team, Wellcome Sanger Institute Scientific Operations: Sequencing Operations, Wellcome Sanger Institute Tree of Life Core Informatics team, Tree of Life Core Informatics collective, Darwin Tree of Life Consortium, and Alex D. Twyford

 “The genome sequence of the European mistletoe, *Viscum album* L. (Santalales: Viscaceae)”

 In this Data Note the authors present a genome assembly from a female specimen of *Viscum album* (European mistletoe; Streptophyta; Magnoliopsida; Santalales; Viscaceae), a scaffolded genome sequence assembly of 94 261.04 megabases mostly (98.67%) into 49 chromosomal pseudomolecules, including the B1 chromosome. Collectively, the findings of the study, the nuclear genome of *Viscum album*, represents a significant advancement because contributes to our understanding on the evolution of parasitic lifestyles in plants. Perhaps a sentence to be added on how the genome of *Viscum album* would improve our understanding of mistletoes biology. However, to strengthen the manuscript, the analysis would benefit from being placed within a broader genomic context by integrating existing organellar genomes and previously published genomic data for this species to enhance the annotation support. Some opportunities for the authors were identified to improve the manuscript in a revision before the manuscript is considered suitable for resubmission.

**Main comments**

  

1. This Data Note reports a chromosome-scale nuclear genome assembly for *Viscum album* generated using PacBio HiFi, 10X assembled reads, Hi-C, and RNA-seq, followed by substantial curation. The dataset is likely to be useful. However, several statements in the Background require factual/citation correction, as explained in detail below, and key methodological/interpretive points (coverage calculations; chromosome/pseudomolecule wording; benchmarking language) need clarification to prevent misinterpretation. We also noted that the PDF of the paper to be reviewed includes public Reader Comments (pp. 13–14) that raise substantive issues; where appropriate, those issues are reiterated here (see below) because they are visible to readers and should be addressed explicitly in a revised version.

 

1. Organelle Genomes. The current manuscript focuses exclusively on the nuclear genome. It is strongly recommended that the authors clarify whether the organelle genomes were also sequenced and assembled as part of their project. If not, a brief justification would be helpful for the reader. If those organelle genomes (chloroplast and mitochondrial) were assembled, their inclusion in the analysis would greatly enrich our understanding of V. album biology. The authors could strengthen their study considerably if these data are acknowledged and integrated, which would, in turn, add robustness to their genome annotation. Moreover, a valuable foundation of prior genomic work on V. album already exists (see Schröder, L., Hohnjec, N., Senkler, M., Senkler, J., Küster, H. and Braun, H.-P. (2022), The gene space of European mistletoe ( *Viscum album*). Plant J, 109: 278-294.(Refer to reference 1) and Schröder L, Rupp O, Senkler M, Rugen N, Hohnjec N, Goesmann A, Küster H and Braun H-P (2023) The *Viscum album* Gene Space database. Front. Plant Sci. 14:1193122. (Refer to reference 1) .

 

1. Clarify the Curation Workflow. The text mentions that data curation was conducted “primarily” in PretextView and HiGlass. To be more precise, it would be helpful to specify if there was a distinct workflow or division of labor between the two tools. For example, you could state: “PretextView was used for the initial contact map scrutiny and scaffolding, while HiGlass was employed for fine-scale border validation and subtle adjustments.” This distinction would give readers a clearer understanding of how each tool contributed to the final assembly.

 

1. Coverage statement appears internally inconsistent. Please verify the coverage calculations. There is a discrepancy that must be checked, the text reports 6,011.18 Gb of HiFi data. Given the estimated genome size of ~91.71 Gb, this would yield approximately 65× coverage, not the stated 26×. The 26× figure would correspond to only ~2.384 Gb of data. This discrepancy suggests either a miscalculation or confusion. Location: Sequence data / Results text stating “~26× coverage” (PDF pp. 5–6). Please reconcile the stated PacBio HiFi coverage (~26×) with the reported sequencing yield and the estimated haploid genome size. If “26×” refers to post-filtered usable bases, define the filtering criteria and report the post-filter yield actually used for assembly. As written, the numbers suggest a substantially higher raw yield/coverage.

 

1. The abstract wording (“49 chromosomal pseudomolecules”) can be read as 49 chromosomes. Please clarify explicitly (in Abstract and Results) that the assembly represents 11 chromosomes (10 A + 1 B), and that chromosomes were submitted as 49 INSDC accessions due to per-record length constraints (if that is the reason). This is important because public assembly summaries may display “49 chromosomes,” which will confuse readers. Please Clarify “49 chromosomal pseudomolecules” vs 11 chromosomes (risk of misinterpretation). Location: Abstract (PDF p. 1) and Results/Assembly description (PDF pp. 6–7; Table 3).

 

1. The manuscript reports an excellent contiguity/QV profile, but the primary assembly shows lower BUSCO/ *k*-mer completeness than the stated benchmark thresholds (while the combined haplotypes are high). Please ( *i*) define precisely what you mean by “meets the recommended reference standard,” and ( *ii*) explain why BUSCO/ *k*-mer behave differently for the primary assembly (e.g., primary vs combined; biology; limitations of the chosen assessment). Revise wording to avoid over-claiming. Benchmarking language (“meets recommended reference standard”) should be qualified. Location: Table 4 and adjacent text (PDF p. 9).

 

1. Scope clarification: organelle genomes and prior *V. album* genomic resources (align with Reader Comment). Please state explicitly whether plastid and mitochondrial genomes were recovered/assembled and deposited. If not, say so and explain why (even briefly). Also, please cite and contextualize the most relevant prior *V. album* resources that are directly useful for annotation/comparative work (e.g., organelle genome work and the “Gene Space” resources). Location: Background and/or Data availability (PDF pp. 3 and 10).

 

1. Background: correct/qualify statements already flagged by Reader Comments. The following statements require verification and alignment between claim and citation: (a) Distribution/trends in Britain (ensure the cited source supports the precise statement; revise if needed). (b) Yield impact (“up to 50%”): either narrow to what the cited study supports (context-specific) or add broader/recent synthesis. (c) Bird “immunity” to viscumin: “immune” is too strong unless directly supported; re-check the cited paper and rephrase conservatively (tolerance/limited sensitivity) with appropriate citation(s). Location: Background (PDF p. 3). In general, the background information on the natural history of *Viscum album* needs to be updated and improved according to comments and available information.

 

1. Methods: flow cytometry text is incomplete / not reproducible as written. The sentence describing nuclei isolation appears grammatically incomplete and does not clearly specify the buffer/protocol used. Please correct the sentence and provide the minimum details needed for reproducibility (buffer name/composition or an unambiguous protocol identifier, and any modifications). Location: Flow cytometry section (PDF p. 3).

 

1. Methods: DNA barcoding is underspecified. “Standard barcode markers” is too generic. Please specify which loci were amplified/sequenced (e.g., rbcL/matK/ITS, as applicable), and provide primer/protocol identifiers and matching/threshold criteria (GenBank/BOLD), to support traceability of specimen identity. Location: DNA barcoding section (PDF pp. 3–4).

 

1. RNA-seq provenance relative to the genome specimen. Please clarify whether RNA-seq derives from the same individual as the genome specimen. If RNA-seq is from a different specimen/tissue, state this explicitly and justify briefly, noting any implications for annotation. Location: Nucleic acid extraction / Table 1 (PDF pp. 4 and 6).

 

1. Methods. Sample acquisition. Why it is important to indicate that the sampled individual plant was a female plant? Would you expect a different result if the sampled individual was a male plant?

**Minor concerns**

  

1. Terminology: “10 autosomes and the B chromosome”. Consider revising to “10 A chromosomes plus one B chromosome” and add a short note that B chromosomes are supernumerary and can vary among individuals/populations, so users interpret this assembly correctly. Location: Assembly description (PDF p. 6).

 

1. Reference list / software list formatting. The “Software Version Source” block appears misplaced/duplicated relative to the bibliographic references. Please separate software reporting from the reference list and format both consistently. Location: Start of References (PDF p. 11).

 

1. Grammar: “that connect via a haustorium” > “that connects via a haustorium.”

 

1. Wording/typo: “collected from on planted trees” > “collected from planted trees” (or equivalent).

 

1. Units/style: standardize Mb/Gb (and avoid ambiguous “megabases” phrasing); state the total length once clearly, then use one unit consistently.

 

1. Table 3 readability: clarify the chromosome-part naming convention (e.g., “Chr5_part1/part2”) so the split across accessions is immediately interpretable.

**Suggested references (if not already included)**

 Briggs (2021), British & Irish Botany (status/trends in Britain & Ireland). DOI: 10.33928/bib.2021.03.419.

 Petersen et al. (2015), Scientific Reports (mitochondrial genome features/gene loss). DOI: 10.1038/srep17588.

 Schröder et al. (2022), The Plant Journal (gene space). DOI: 10.1111/tpj.15558.

 Schröder et al. (2023), Frontiers in Plant Science (Gene Space database). DOI: 10.3389/fpls.2023.1193122.

 (Optional, for nuanced resource acquisition language) Thomas et al. (2023) indicates discussion that mistletoe extracts at least some carbon from the host.

 (Optional, for host performance under stress) González de Andrés et al. (2024), Tree Physiology, on mistletoe impacts on tree carbon/water/nutrients under environmental stress.

**Recommendation**

 Approved with Reservations. The dataset is likely sound and useful, but accuracy, clarity, and reproducibility require the revisions above, particularly ( *i*) chromosome/pseudomolecule wording, ( *ii*) coverage calculation transparency, ( *iii*) qualified benchmarking language, and ( *iv*) corrected Background claims with appropriate citations.

Are sufficient details of methods and materials provided to allow replication by others?

No

Is the rationale for creating the dataset(s) clearly described?

Partly

Are the datasets clearly presented in a useable and accessible format?

Partly

Are the protocols appropriate and is the work technically sound?

Partly

Reviewer Expertise:

Phylogeography, plant-hummingbird interactions, mistletoes, genomics

We confirm that we have read this submission and believe that we have an appropriate level of expertise to confirm that it is of an acceptable scientific standard, however we have significant reservations, as outlined above.

## References

[1] : The gene space of European mistletoe (Viscum album ). *The Plant Journal* .2022;109(1) : 10.1111/tpj.15558 278-294 10.1111/tpj.15558 34713513

[2] : The Viscum album Gene Space database. *Frontiers in Plant Science* .2023;14: 10.3389/fpls.2023.1193122 10.3389/fpls.2023.1193122 PMC1035972837484460

[3] : Mistletoe, Viscum album (Santalaceae), in Britain and Ireland; a discussion and review of current status and trends. *British & Irish Botany* .2021;3(4) : 10.33928/bib.2021.03.419 10.33928/bib.2021.03.419

[4] : Massive gene loss in mistletoe (Viscum, Viscaceae) mitochondria. *Scientific Reports* .2015;5(1) : 10.1038/srep17588 10.1038/srep17588 PMC466725026625950
